# Awareness of Gestational Diabetes Mellitus Among Women in the Al-Baha Region, Saudi Arabia

**DOI:** 10.7759/cureus.50163

**Published:** 2023-12-08

**Authors:** Tajelsir M Ali, Eman A Keshk, Osama M Almaqadi, Khader M Alsawlihah, Mohammed M Alzahrani, Ahmed A Alzahrani, Abdullah Y Alsalhi, Saeed M Alzahrani, Jamaan A Alzahrani, Muteb A Alzahrani

**Affiliations:** 1 Obstetrics and Gynaecology, Faculty of Medicine, Al-Baha University, Al-Baha, SAU; 2 Faculty of Medicine, Suez Canal University, Ismailia, EGY; 3 Medicine, Al-Baha University, Al-Baha, SAU

**Keywords:** gestational diabetes mellitus (gdm), kingdom of saudi arabia (ksa), al-baha region, women, awareness

## Abstract

Background

Gestational diabetes mellitus (GDM) is a condition characterized by impaired glucose tolerance that develops during pregnancy. The prevalence of GDM is increasing globally, including in the Al-Baha region of Saudi Arabia. However, there needs to be more data on the awareness of women in this region regarding GDM and its associated risks. This research aimed to evaluate the level of awareness among women in the Al-Baha region regarding GDM.

Methodology

This study followed an observational cross-sectional design conducted from April 2023 to December 2023. A simple random sampling technique was used to select 457 participants from the resident women of reproductive age in the Al-Baha region. Data were collected through a self-administered questionnaire that assessed knowledge and awareness of GDM risk factors, assessment, therapy, and implications. The questionnaire included a 12-item section evaluating GDM awareness, with correct answers receiving a score of 1. Descriptive statistics were used to analyze the data with Statistical Product and Service Solutions (SPSS, version 28) (IBM SPSS Statistics for Windows, Armonk, NY).

Results

The majority of participants fell into the age group of more than 36 years (n=207, 45.3%), with a significant proportion having completed university/diploma education (n=282, 61.7%), and most of them worked outside the health sector (n=283, 61.9%). Approximately 27.8% correctly identified that the number of pregnancies does not increase the chance of developing GDM. Only (n=48, 10.5%) accurately identified the usual time for diagnosing GDM in the absence of risk factors, which is between weeks 24 and 28 of pregnancy. Similarly, 26.0% (119 participants) correctly recognized a history of a previous pregnancy with a child weighing more than 4.5 kg as a factor that increases the suspicion of developing GDM in the future. However, it is important to note that the majority of participants (n=311, 68.1%) had a poor level of awareness regarding GDM.

Conclusion

The findings revealed that the overall level of knowledge about GDM was poor, with less than 10% of participants demonstrating adequate awareness. The study also highlighted that over 80% of the participants were unaware of GDM.

## Introduction

Diabetes mellitus is characterized by persistent hyperglycemia and changes in carbohydrates, fats, and protein metabolism as a result of problems with insulin secretion and insulin resistance [1]. An impairment in glucose tolerance that first appears during pregnancy is known as gestational diabetes [2]. The prevalence of gestational diabetes mellitus (GDM), which ranges from 1% to 20% globally, is increasing especially among African, Hispanic, Indian, and Asian women than Caucasian women [3,4]. The prevalence of GDM has increased by two to three times recently, ranging from 8.9% to 53.4% [5-7]. The prevalence of GDM in Saudi Arabia was 11.7% [7]. Moreover, a recent study reported that the prevalence of women with a previous history of GDM was 15.3% in the Al-Qassim region. The level of awareness regarding GDM was poor among 60.3% of women [8]. GDM incidence is increasing worldwide because of progressing trends in obesity and the advancement of maternal age among women during childbearing age [1]. GDM is associated with adverse fetal-maternal outcomes, such as preeclampsia, preterm birth, fetal macrosomia, polyhydramnios, shoulder dystocia, Caesarean section, neonatal respiratory distress, neonatal hypoglycemia, and perinatal mortality. Appropriate management of this disorder is crucial for a favorable pregnancy outcome [9].

Limited data are available regarding women's awareness of GDM [10-12]. Considering the high prevalence of this disorder in our population, it emerges as a crucial endeavor to realize effective post-diagnosis counseling with the aim of spreading female awareness of the fetal-maternal risks related to it. Therefore, we aim to evaluate women's awareness of GDM in the Al-Baha region, Saudi Arabia.

## Materials and methods

Objectives

In this study, the aim was to evaluate the awareness of women in the Al-Baha region toward GDM. The specific objectives were to determine their awareness of the risk factors and complications associated with GDM.

Study design

An observational cross-sectional study design was utilized for this research. The study was conducted from April 2023 to November 2023 in the Al-Baha region, Saudi Arabia.

Sampling technique and sample size

The study population consisted of resident women in the Al-Baha region. Participants were selected using a simple random sampling technique. The sample size was calculated using the EPI info program (Centers for Disease Control and Prevention, Atlanta, Georgia), considering a 95% confidence interval, a 5% margin of error, and the total population of Al-Baha. The estimated sample size was 422, accounting for a 10% non-response rate.

Study participants

The inclusion criteria for the study were Al-Baha resident women of reproductive age (18-45 years). Women who were unwilling to participate or fell outside the reproductive age range were excluded from the study.

Data collection and instrumentation/data collection method

A self-administered questionnaire, adapted from a previous study, was distributed among women using an electronic format [8]. The survey aimed to collect socio-demographic information and assess knowledge and awareness regarding diabetes mellitus (DM) and GDM risk factors, evaluation, treatment, and consequences. For the evaluation of GDM awareness, a 12-item questionnaire was employed, where a value of 1 was assigned to correct answers and a value of 0 to incorrect answers. Questions #6, #7, and #9 allowed for multiple responses, resulting in a total awareness score of 19 items. The overall awareness score ranged from 1 to 19 points. Participants were categorized based on their level of awareness, with scores below 50% indicating poor awareness, scores between 50% and 75% indicating moderate awareness, and scores above 75% indicating good awareness.

Pilot study

A pilot study was conducted on 20 participants to test the suitability and clarity of the questionnaire and to estimate the time required for data collection.

Data analysis

The data were analyzed using Statistical Package for the Social Sciences (SPSS, version 28) (IBM Corporation, Armonk, NY). Descriptive statistics were performed using numbers and percentages.

Ethical consideration

The research study, approved under the reference number REC/OB/BU-FM/2023/15, obtained ethical approval and was closely monitored by the Al-Baha University Research Committee to ensure compliance with ethical guidelines. Participants actively provided informed consent, and measures were taken to protect their privacy throughout the study. This research carried no known risks associated with the participation.

## Results

Sociodemographic data

Table 1 reveals the characteristics of the study participants. The majority of participants fell into the age group of more than 36 years (45.3%), followed by 26-35 years (30.2%) and 18-25 years (24.5%). A significant proportion had completed university/diploma education (61.7%), while high school education was reported by 25.2% of participants. The employment status showed that most of the participants worked outside the health sector (61.9%).

**Table 1:** Sociodemographic data (n=457).

Sociodemographic Data		Count	%
Age Grouping	18-25 Years	112	24.5%
	26-35 Years	138	30.2%
	More Than 36 Years	207	45.3%
Educational Status	Primary	17	3.7%
	Intermediate	15	3.3%
	High School	115	25.2%
	University/Diploma	282	61.7%
	Postgraduate Degree	28	6.1%
Employment Status	Housewife	29	6.3%
	Work Within the Health Sector	145	31.7%
	Work Outside the Health Sector	283	61.9%
Associated Chronic Disease	No	332	72.6%
	Yes	125	27.4%
Specific Chronic Disease	DM	45	9.8%
	HTN	32	7.0%
	Hypothyroidism	8	1.8%
	Hyperlipidemia	40	8.8%
Pregnancy Times	None	107	23.4%
	1-2	114	24.9%
	3-5	167	36.5%
	>5	69	15.1%
Have you ever had what is called (gestational diabetes)?	No	394	86.2%
	Yes	63	13.8%
Do you know anyone who has had gestational diabetes?	No	165	36.1%
	Yes	292	63.9%

In terms of associated chronic diseases, 27.4% reported having at least one chronic disease. The most prevalent specific chronic diseases were DM (9.8%) and hyperlipidemia (8.8%). Regarding pregnancy history, the highest proportion of participants reported experiencing three to five pregnancies (36.5%). Most participants had never experienced gestational diabetes (86.2%), although a notable percentage reported knowing someone who had (63.9%).

Assessment of awareness regarding GDM

The results of the research reveal that a considerable number of participants demonstrated accurate knowledge regarding certain aspects related to GDM. For instance, 27.8% (127 participants) correctly identified that increasing the number of pregnancies does not increase the chance of developing GDM. Additionally, 61.1% (279 participants) recognized that women with a previous history of GDM have a higher chance of developing it again. Moreover, 49.9% (228 participants) correctly acknowledged that women with a family history of diabetes are at a higher risk of developing GDM. Furthermore, only 10.5% (48 participants) accurately identified the usual time to diagnose GDM in the absence of risk factors as weeks 24-28. Among the participants, 40.9% (187 participants) correctly identified blood analysis after drinking a glucose solution as the appropriate analysis for diagnosing GDM. In terms of treatment for GDM, 61.3% (280 participants) correctly recognized the importance of organizing meals and exercise. Additionally, 33.7% (154 participants) correctly acknowledged the necessity of analyzing blood after drinking glucose 6-12 weeks after childbirth for those who developed GDM to ensure its removal. Lastly, 26.0% (119 participants) correctly identified a history of a previous pregnancy with a child weighing more than 4.5 kg as a factor that increases the suspicion of developing GDM in the future.

**Figure 1:**
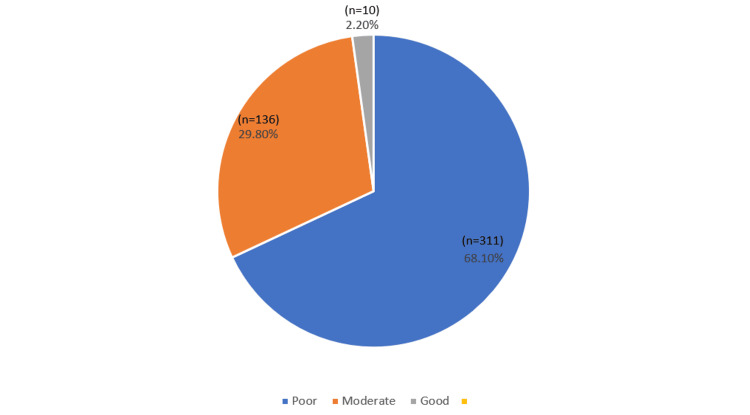
Overall Awareness level among the study participants.

Moreover, 27.1% (124 participants) correctly recognized that weight gain before pregnancy or its rapid increase during the first months of pregnancy may contribute to the development of GDM. Furthermore, 19.3% (88 participants) correctly identified that polycystic ovary syndrome (PCOS) increases the risk of GDM. Additionally, 26.9% (123 participants) correctly acknowledged that a mother's age over 35 years old may contribute to GDM. It is important to note that the majority of participants had a poor awareness level, with 68.1% (311 participants) falling into this category. Table 2 and Figure 1 present the overall awareness level among the study participants.

**Table 2:** Assessment of awareness regarding GDM. * Indicates the correct answer. † Variable with multiple response answers.

Awareness Assessment		Count	%
Increasing the number of pregnancies increases the chance of developing GDM	No*	127	27.8%
	Yes	106	23.2%
	I don't know	224	49.0%
Women previous history of GDM have a higher chance of developing it again	No	43	9.4%
	Yes*	279	61.1%
	I don't know	135	29.5%
Woman with a family history of diabetes has a higher chance of developing GDM	No	92	20.1%
	Yes*	228	49.9%
	I don't know	137	30.0%
Usual time to diagnose GDM in the absence of risk factors	Week 12-17	60	13.1%
	Week 18-23	99	21.7%
	Week 24-28*	48	10.5%
	I don't know	250	54.7%
Analysis used to diagnose GDM†	I don't know	128	28.0%
	Urinalysis	129	28.2%
	Blood analysis	316	69.1%
	Blood analysis after drinking a glucose solution*	187	40.9%
Treatment used for a person with GDM†	I don't know	117	25.6%
	Organizing meals and exercise *	280	61.3%
	Insulin injection *	131	28.7%
	Taking diabetes medications orally *	112	24.5%
GDM is:†	I don't know	99	21.7%
	It usually disappears after birth *	261	57.1%
	It can affect the unborn baby if gestational diabetes is not treated *	197	43.1%
	A woman with gestational diabetes may develop diabetes mellitus II in the future *	204	44.6%
Necessary to analyze the blood after drinking glucose 6-12 weeks after childbirth for those who developed GDM to ensure its removal	No	39	8.5%
	Yes*	154	33.7%
	I don't know	264	57.8%
Having a previous pregnancy history for the following cases increases the suspicion of developing GDM in the future †	I don't know	287	62.8%
	History of a previous pregnancy with a child weighing more than 4.5 kg *	119	26.0%
	Prior pregnancy history of a stillborn baby *	59	12.9%
	Previous history of pregnancy accompanied by excess fluid in the fetus *	92	20.1%
	Prior history of recurrent vaginal or urinary symptoms during pregnancy *	76	16.6%
Weight gain before pregnancy or its rapid increase during pregnancy in the first months may contribute to GDM	No	34	7.4%
	Yes *	124	27.1%
	I don't know	101	22.1%
	Maybe	198	43.3%
PCOS increases the risk of GDM	No	64	14.0%
	Yes*	88	19.3%
	I don't know	176	38.5%
	I don't know what the syndrome is	129	28.2%
A mother's age over 35 years old may contribute to GDM	No	62	13.6%
	Yes*	123	26.9%
	I don't know	115	25.2%
	Maybe	157	34.4%
Total awareness score (Mean ± SD)	8.1 ± 3.1		

## Discussion

GDM is a pregnancy illness that harms the health of many millions of women globally [13]. It is a common metabolic illness that can affect approximately 25% of pregnant women, and the global prevalence ranges between 5% and 25.5% [14]. GDM refers to any level of glucose intolerance that occurs during pregnancy due to insulin resistance and pancreatic cell malfunction [15]. It is an illness that occurs during the second and third trimesters of pregnancy and features marked insulin resistance secondary to placental hormonal release. GDM also features urgent pregnancy problems such as extra fetal growth and adiposity, with the consequent threat of delivery strain and hypertensive ailments in pregnancy [16]. While the cause of GDM is not generally unknown, some theories consider obesity, late parental age, and women from specific races as high risks [17]. Knowing the public's knowledge and perception of GDM is vital in designing and implementing proper preventative and management strategies. This discussion examines the knowledge and views about GDM held by participants in research performed in the Al-Baha region of Saudi Arabia.

Generally, the study outcomes indicate that the participants had a poor understanding of GDM. A large percentage of respondents (68.1%) had a poor level of awareness, 29.8% had reasonable awareness levels, and only 2.2% had a good level of awareness of the disease. These outcomes resemble recent research performed in Saudi Arabia, where 60.3% of participants had poor awareness levels, 33% reported moderate awareness, and only 6.6% reported a good level of awareness [8]. Another study surpassed the current findings. Based on the study outcomes, 77.8% of women in Jeddah reported poor knowledge, and only 6.1% understood GDM properly [18]. Additionally, 93.69% of participants reported poor awareness of GDM [19]. This study represents extreme levels of poor awareness compared to other studies in Saudi Arabia that reported fair awareness. Awareness of GDM was poor among respondents (53.45%), with only a marginal percentage of 7.80% knowing GDM [20].

Alnaeem [21] reported that 45.2% of participants were unaware of GDM. Alnaim [22] presented outcomes about GDM awareness in Saudi Arabia, with most respondents (50.5%) having fair knowledge, 13% not knowledgeable, and 36.5% fully aware of GDM. Moreover, Hakim et al. [23] discovered that most respondents knew GDM, with 53.6% of participants indicating that they had heard about the disease, 35.2% partially heard, and only 11.2% reported not hearing about GDM. It is the only study with participants above 50% with positive knowledge concerning GDM. This calls for proper awareness and education on GMD among women in Saudi Arabia. However, in the context of the impacts of GDM, numerous studies showed positive results. The risk to normal delivery was the most cited consequence of GDM [19,21].

The study findings also indicated that most participants have never had gestational diabetes (86.2%), with only 13.8% reporting hearing about the disease. These outcomes are inconsistent with other research about Saudi Arabia. Past studies, such as [24], indicate increased dominance of GDM in Saudi Arabia. The authors attribute these higher numbers to the increased occurrence of type 2 diabetes, obesity, and the custom of Saud women conceiving in old age. The dominance of GDM was previously reported at 12.5%, but current studies reported a higher percentage of GDM among Saudi women at 36.6% [25]. This shows an increasing trend in the occurrence of GDM, as indicated in other past research [26,27]. However, one study indicates a lower trend in the occurrence of GDM among Saudi women. A study reported a 24% prevalence [26]. This might indicate that massive amounts of data were missing from this study, and it might have included a mixture of Saudi women and foreigners. Research also shows that Saudi Arabia is among the top 10 nations with an increased occurrence of diabetes type 2 at 18.3%, and it is forecast to be higher by 2045 [28]. This might be the foundation of the increased cases of GDM among Saudi women. Therefore, effective preventive and management measures, particularly screening, should be operationalized to help Saudi women understand their status earlier.

Although the study indicates that most participants have never had the disease, most participants reported knowing people diagnosed with the disease at 63.9%, with approximately 36.1% not knowing anyone with the disease. This is consistent with a study where 75% of participants understood that risk factors linked to GDM, such as obesity, eating fast foods, and stress exposed women to GDM. Research also shows that participants who knew people diagnosed with GDM or had family members were more knowledgeable about the disease [21]. This shows that knowing people diagnosed with the disease increases disease awareness.

The study outcomes also indicate that a larger percentage of participants (61.7%) had a university or diploma, 25.2% are high school, 6.1% had a postgraduate degree, 3.7% had primary education, and 3.3% had intermediate education status. This indicates that a larger portion of the participants were knowledgeable. This is consistent with a current study where the majority of the participants (47.9%) reported higher education levels, 35.3% had a secondary level of education, 9.4% had intermediate education, and 5.4% had primary education [21]. Irrespective of the highest education levels in both studies, GDM awareness remained low. This shows that GDM awareness and knowledge are not aligned with the country’s education system or knowledge stream. A current study also used participants, with more than 65.1% having a university education level. In all these studies, participants with university education levels and above scored a higher knowledge level on GDM. Additionally, research indicates that participants with higher education were much more aware compared to others.

In the context of employment status, a large percentage of participants - 61.9% - operated outside the health sector, 31.7% operated within the health sector, and 6.3% were housewives. The huge percentage of participants operating outside of healthcare might be the reason for low awareness rates. In studies where a large percentage of participants (86% were housewives) and only 13% were employed, the knowledge gap is minimal compared to the current study [21]. This might indicate that employment is not a significant factor in knowing GDM. However, these employed people must be educated. This implies that, if educated people presented better knowledge of GDM, then employed people, particularly in the health sector, are more knowledgeable on GDM than others.

Furthermore, the study outcomes indicate that most participants (54.7%) do not know the usual time to diagnose GDM when risk factors are unavailable. This indicates the importance of risk factors as examined in diverse studies. A study discovered that 75% of participants understood threats causing GDM, such as consumption of unhealthy foods, obesity, and depression, but 45.2% lacked knowledge with no family histories [21].

Limitations

The study has several limitations that should be taken into consideration when interpreting the findings. Firstly, the generalizability of the results may be limited since the sample was selected using a simple random sampling technique and may only partially represent part of the population of women in the Al-Baha region or other regions. Secondly, the reliance on self-reported data introduces the possibility of recall bias or social desirability bias, potentially impacting the accuracy and reliability of the responses.

## Conclusions

In conclusion, this study analyzes the public's awareness and perception of GDM in Saudi Arabia. The overall level of knowledge was poor and fell below 10%. The levels of unawareness peaked at over 80%. This indicates that the knowledge transmission on GDM is ineffective, and most people do not understand GDM. The major determinant factors include education levels, employment status, and past history with the disease. Educated people and those with a history of the disease are more knowledgeable than others. However, irrespective of the higher education and employment levels, the study discovers a gap in GDM awareness.

This study underscores targeted education programs, particularly for the uneducated and housewives who primarily stay at home. Medical professionals are obligated to share precise information, and medical authorities should ensure the public gets accurate information about GDM. By expanding awareness and understanding of GDM, it will be easy to introduce preventative programs, inspire timely detection, and improve the general management of this pregnancy disease.
